# Supplementary material for: The causal role of circulating inflammatory markers in osteoporosis: a bidirectional Mendelian randomized study
Source: Front Immunol. 2024 Jul 18;15:1412298. doi: 10.3389/fimmu.2024.1412298 (PMC11291241; doi:10.3389/fimmu.2024.1412298)
Supplement: Supplementary file 1 [file DataSheet_1.zip › supplementary material/OSTEOPOROSIS-CIM/GCST90274782.tsv.gz_forest.pdf]

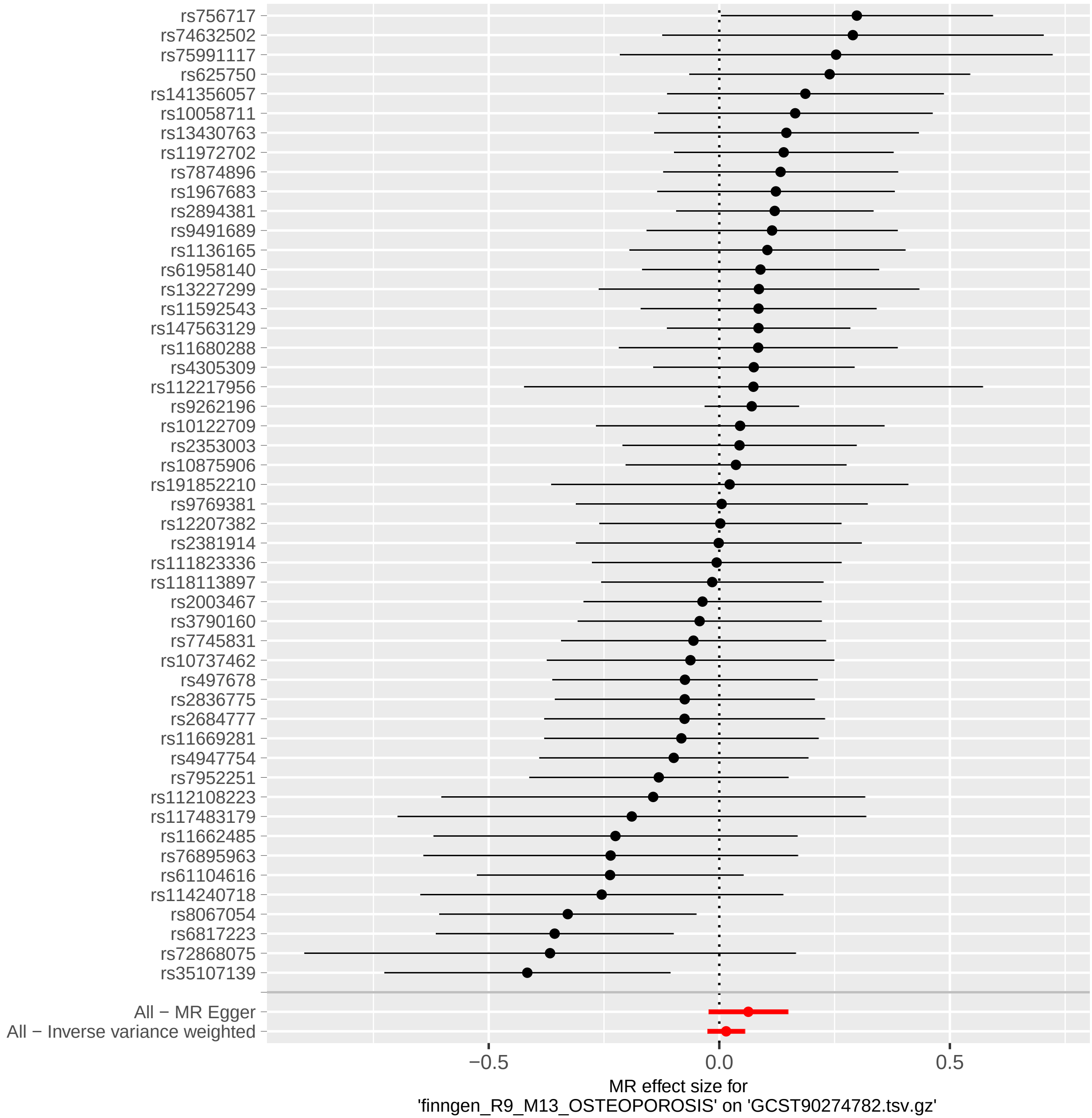
rs756717
rs74632502
rs75991117
rs625750
rs141356057
rs10058711
rs13430763
rs11972702
rs7874896
rs1967683
rs2894381
rs9491689
rs1136165
rs61958140
rs13227299
rs11592543
rs147563129
rs11680288
rs4305309
rs112217956
rs9262196
rs10122709
rs2353003
rs10875906
rs191852210
rs9769381
rs12207382
rs2381914
rs111823336
rs118113897
rs2003467
rs3790160
rs7745831
rs10737462
rs497678
rs2836775
rs2684777
rs11669281
rs4947754
rs7952251
rs112108223
rs117483179
rs11662485
rs76895963
rs61104616
rs114240718
rs8067054
rs6817223
rs72868075
rs35107139
All − MR Egger
All − Inverse variance weighted
−0.5
0.0
0.5
MR effect size for
'finngen_R9_M13_OSTEOPOROSIS' on 'GCST90274782.tsv.gz'
